# Supplementary material for: Psychopathological Impact in Patients with History of Rheumatic Fever with or without Sydenham’s Chorea: A Multicenter Prospective Study
Source: Int J Environ Res Public Health. 2022 Aug 25;19(17):10586. doi: 10.3390/ijerph191710586 (PMC9517806; doi:10.3390/ijerph191710586)
Supplement: Supplementary file 1 [file ijerph-19-10586-s001.zip › ijerph-1862140-supplementary.pdf]

International Journal of
Environmental Research and Public Health

MDPI

Table S1

WSAS across Chorea
Independent-Samples Mann-Whitney U Test Summary

Total Number **Copyright:** © 2022 by the authors. Licensee MDPI, Basel, Switzerland. This article is an open access article distributed under the terms and conditions of the Creative Commons Attribution (CC BY) license (https://creativecommons.org/licenses/by/4.0/).	48
Mann-Whitney U	371.500
Wilcoxon W	602.500
Test Statistic	371.500
Standard Error	38.040
Standardized Test Statistic	2.313
Asymptotic Significance (2-sided test)	0.021

Table S2

Hypothesis Test Summary

	Null hypothesis	Test	Significance
1	The distribution of WSAS is the same across categories of Chorea	Independent-Samples Mann-Whitney U Test	0.021
2	The distribution of WSAS 1 is the same across categories of Chorea	Independent-Samples Mann-Whitney U Test	0.740
3	The distribution of WSAS 2 is the same across categories of Chorea	Independent-Samples Mann-Whitney U Test	0.158
4	The distribution of WSAS 3 is the same across categories of Chorea	Independent-Samples Mann-Whitney U Test	0.028
5	The distribution of WSAS 4 is the same across categories of Chorea	Independent-Samples Mann-Whitney U Test	0.133
6	The distribution of WSAS 5 is the same across categories of Chorea	Independent-Samples Mann-Whitney U Test	0.017

Table S3: A, B

Independent-Samples Mann-Whitney U Test Summary

Total Number	48
Mann-Whitney U	255.000
Wilcoxon W	426.000
Test Statistic	255.000
Standard Error	32.822
Standardized Test Statistic	2.285
Asymptotic Significance (2-sided test)	0.022
Exact Significance (2-sided test)	0.028

Independent-Samples Mann-Whitney U Test Summary

Total Number	48
Mann-Whitney U	260.500
Wilcoxon W	431.500
Test Statistic	260.500
Standard Error	32.900
Standardized Test Statistic	2.447
Asymptotic Significance (2-sided test)	0.014
Exact Significance (2-sided test)	0.017

Table S4: A, B, C, D

GAD-7 across Chorea
Independent-Samples Mann-Whitney U Test Summary

Total Number	48
Mann-Whitney U	337.000
Wilcoxon W	568.000
Test Statistic	337.000
Standard Error	45.997
Standardized Test Statistic	1.163
Asymptotic Significance (2-sided test)	0.245

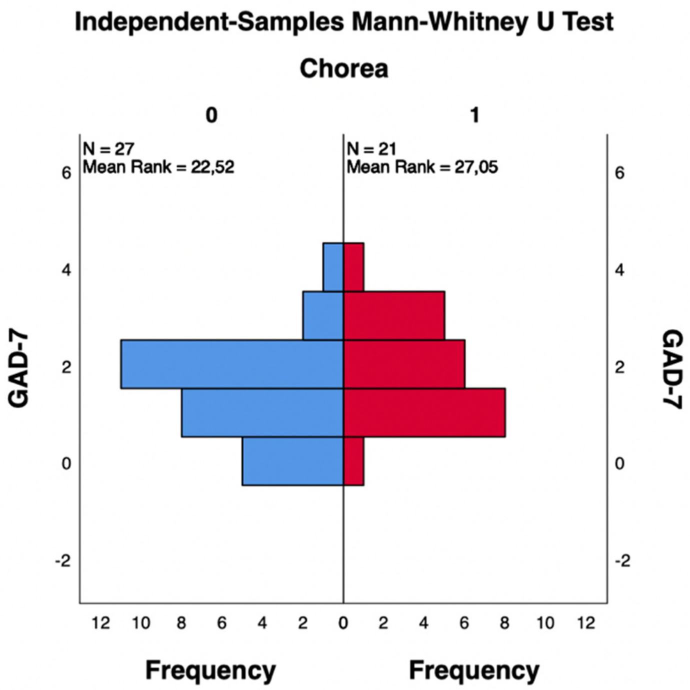

DSM-5 Level 2 – Anxiety across Chorea
Independent-Samples Mann-Whitney U Test Summary

Total Number	47
Mann-Whitney U	275.000
Wilcoxon W	506.000
Test Statistic	275.000
Standard Error	45.562
Standardized Test Statistic	0.048
Asymptotic Significance (2-sided test)	0.962

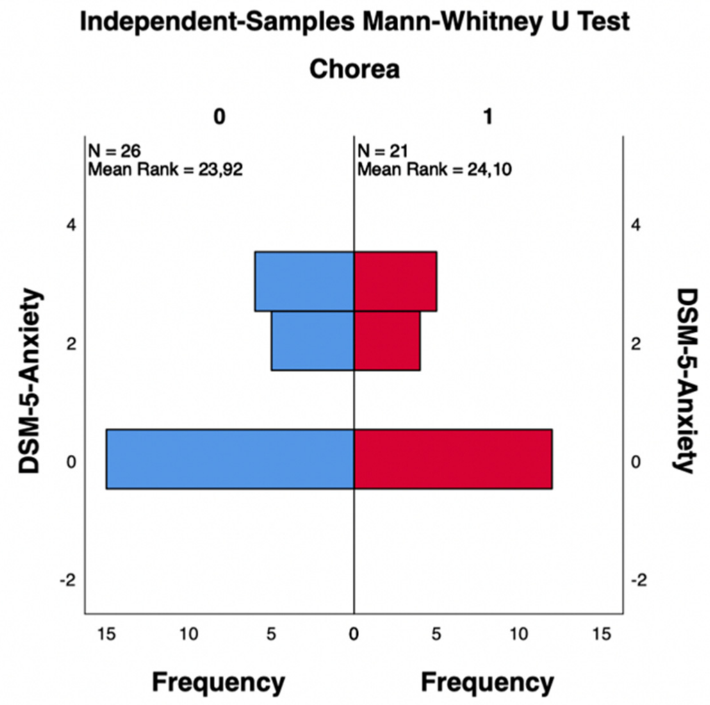

Table S5: A, B, C, D

PHQ-9 across Chorea
Independent-Samples Mann-Whitney U Test Summary

Total Number	48
Mann-Whitney U	317.000
Wilcoxon W	548.000
Test Statistic	317.000
Standard Error	44.924
Standardized Test Statistic	0.746
Asymptotic Significance (2-sided test)	0.456

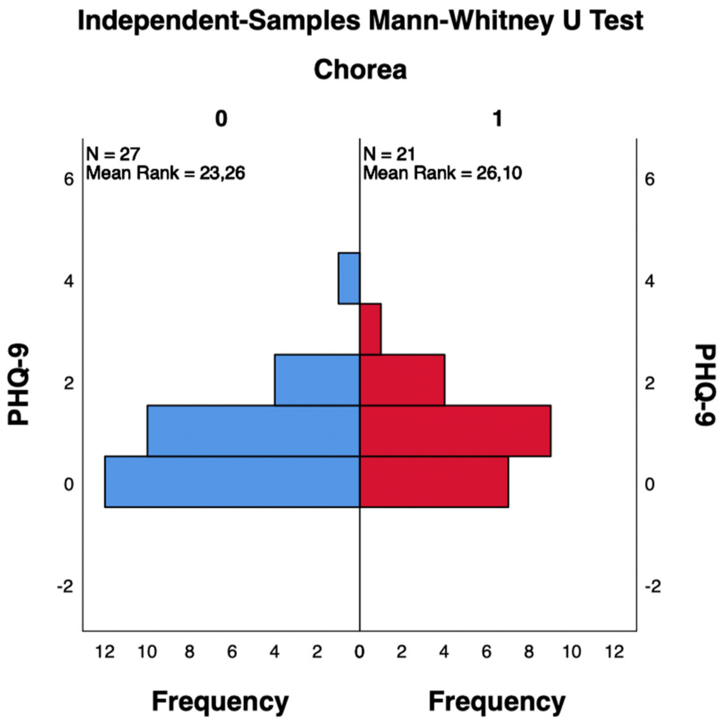

DSM-5 Level 1 – Depression across Chorea
Independent-Samples Mann-Whitney U Test Summary

Total Number	48
Mann-Whitney U	270.000
Wilcoxon W	501.000
Test Statistic	270.000
Standard Error	37.439
Standardized Test Statistic	−0.361
Asymptotic Significance (2-sided test)	0.718

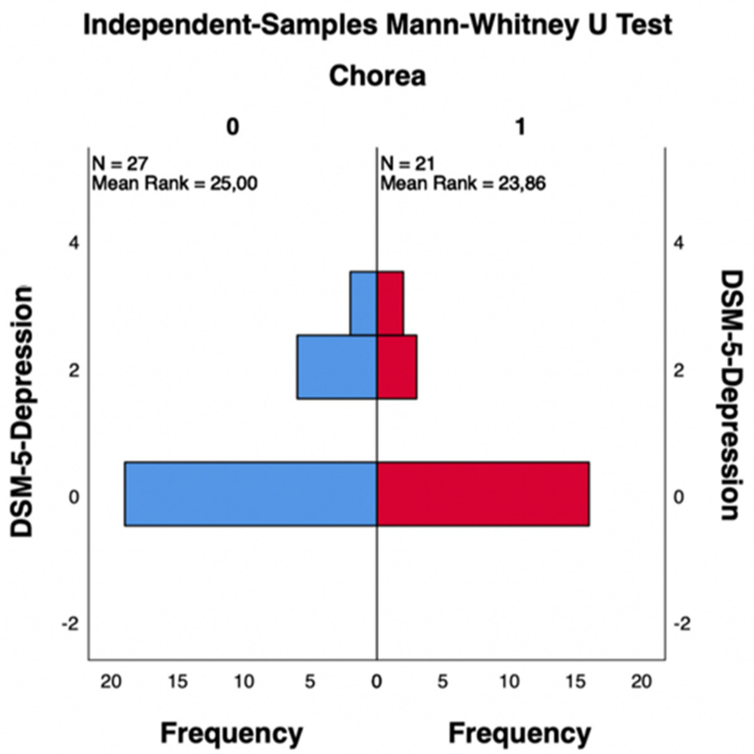
Independent-Samples Mann-Whitney U Test
Chorea
0
1
N = 27
Mean Rank = 25,00
N = 21
Mean Rank = 23,86
DSM-5-Depression
DSM-5-Depression
4
2
0
-2
20 15 10 5 0 5 10 15 20
Frequency
Frequency

Table S6: A, B, C, D, E, F, G, H

Group Statistics

	Chorea	Number	Mean	Standard Deviation	Standard Error Mean
I	0	27	1.22	1.251	0.241
	1	21	1.43	1.076	0.235
II	0	27	1.11	1.086	0.209
	1	21	1.24	0.995	0.217
III	0	27	1.19	1.145	0.220
	1	21	1.62	1.024	0.223
IV	0	27	1.33	1.109	0.214
	1	21	1.86	1.236	0.270
V	0	27	0.52	0.849	0.163
	1	21	0.62	1.024	0.223
VI	0	27	0.19	0.622	0.120
	1	21	0.19	0.602	0.131
VII	0	27	0.15	0.456	0.088
	1	21	0.33	0.796	0.174
VIII	0	27	0.85	1.064	0.205
	1	21	1.24	1.261	0.275
IX	0	27	0.48	0.935	0.180
	1	21	0.33	0.658	0.144
X	0	27	0.48	1.087	0.209
	1	21	0.48	0.873	0.190
XI	0	27	0.26	0.712	0.137
	1	21	0.43	0.870	0.190
XII	0	27	0.81	1.111	0.214
	1	21	1.24	1.261	0.275
XIII	0	27	0.89	1.577	0.304
	1	21	0.62	1.322	0.288

Hypothesis Test Summary

	Null Hypothesis	Test	Significance
1	The distribution of I is the same across categories of Chorea	Independent-Samples Mann-Whitney U Test	0.407
2	The distribution of II is the same across categories of Chorea	Independent-Samples Mann-Whitney U Test	0.595
3	The distribution of III is the same across categories of Chorea	Independent-Samples Mann-Whitney U Test	0.182

4	The distribution of IV is the same across categories of Chorea	Independent-Samples Mann-Whitney U Test	0.119
5	The distribution of V is the same across categories of Chorea	Independent-Samples Mann-Whitney U Test	0.881
6	The distribution of VI is the same across categories of Chorea	Independent-Samples Mann-Whitney U Test	0.891
7	The distribution of VII is the same across categories of Chorea	Independent-Samples Mann-Whitney U Test	0.416
8	The distribution of VIII is the same across categories of Chorea	Independent-Samples Mann-Whitney U Test	0.294
9	The distribution of IX is the same across categories of Chorea	Independent-Samples Mann-Whitney U Test	0.763
10	The distribution of X is the same across categories of Chorea	Independent-Samples Mann-Whitney U Test	0.691
11	The distribution of XI is the same across categories of Chorea	Independent-Samples Mann-Whitney U Test	0.427
12	The distribution of XII is the same across categories of Chorea	Independent-Samples Mann-Whitney U Test	0.179
13	The distribution of XII is the same across categories of Chorea	Independent-Samples Mann-Whitney U Test	0.585

III across Chorea
Independent-Samples Mann-Whitney U Test Summary

Total Number	48
Mann-Whitney U	345.000
Wilcoxon W	576.000
Test Statistic	345.000
Standard Error	46.057
Standardized Test Statistic	1.335
Asymptotic Significance (2-sided test)	0.182

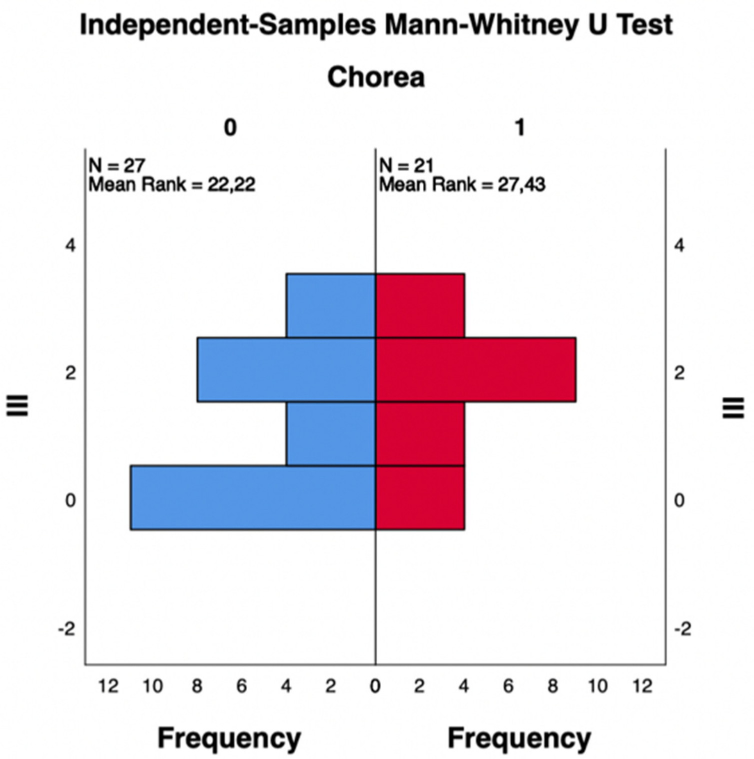

IV across Chorea
Independent-Samples Mann-Whitney U Test Summary

Total Number	48
Mann-Whitney U	356.000
Wilcoxon W	587.000
Test Statistic	356.000
Standard Error	46.490
Standardized Test Statistic	1.559
Asymptotic Significance (2-sided test)	0.119

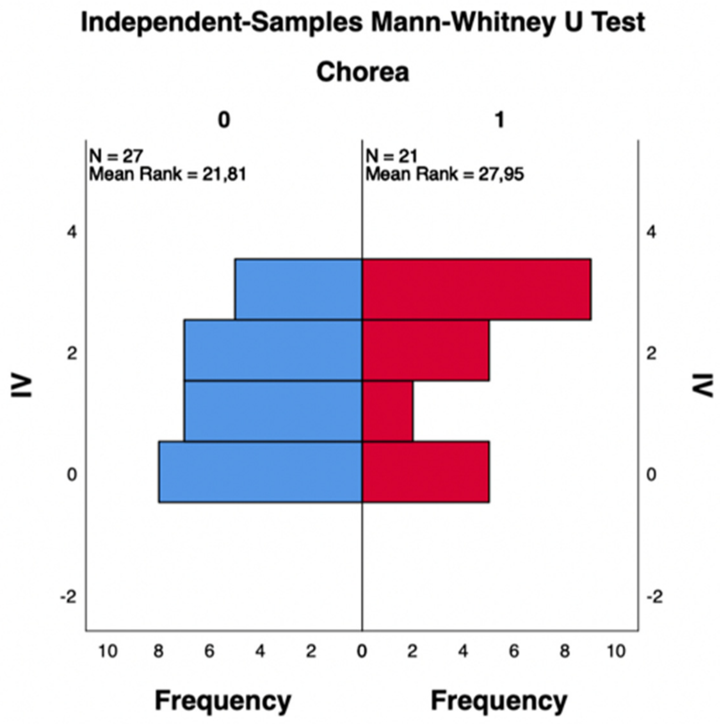

XII across Chorea

Independent-Samples Mann-Whitney U Test Summary

Total Number	48
Mann-Whitney U	344.000
Wilcoxon W	575.000
Test Statistic	344.000
Standard Error	44.986
Standardized Test Statistic	1.345
Asymptotic Significance (2-sided test)	0.179

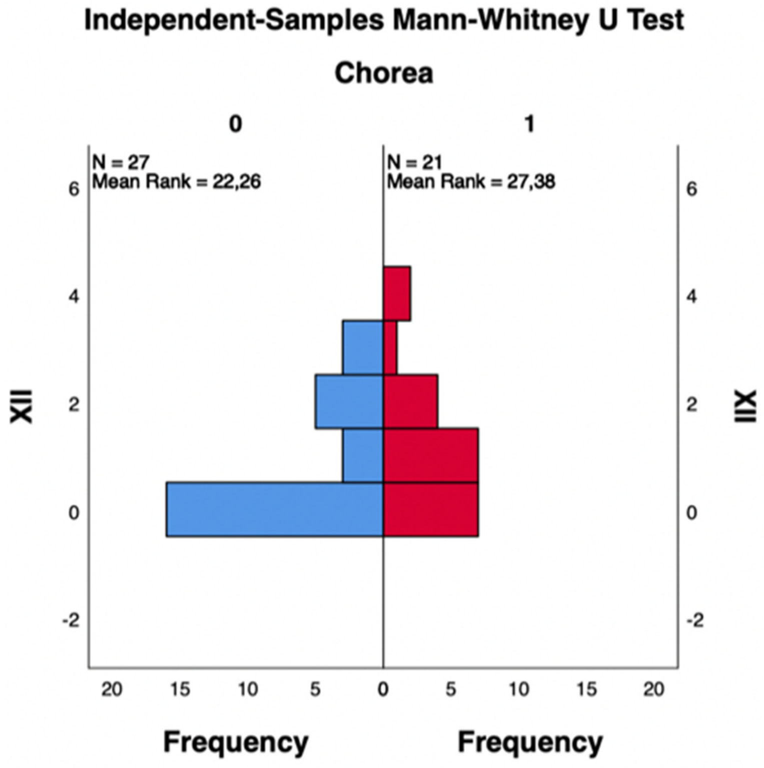

Table S7: A, B

SC patients		
**Paziente**	**Centro di riferimento**	**SCID-5 Discreta**
1	Pisa	Criterianotmet
2	Pisa	Attention-deficit hyperactivity disorder, combined type (F90.2)
3	Pisa	Criterianotmet
4	Bologna	primaryinsomnia (F51.01)
5	Bologna	Post-traumatic stress disorder (PTSD) (Subtype A: B, C, D, E +, A-) (F43.1)
6	Bologna	Panic disorder [episodic paroxysmal anxiety] (F41.0), primary insomnia (F51.01)
7	Bologna	primaryinsomnia (F51.01)
8	Bologna	Panic disorder [episodic paroxysmal anxiety] (F41.0), Post-traumatic stress disorder (PTSD) (F43.1)
9	Bologna	Major depressive disorder, single episode, in full remission (F32.5), Other eating disorders (F50.8)
10	Bologna	Otherspecifiedphobia (F40.298)
11	Bologna	Major depressive disorder, recurrent, mild (F33.0)
12	Bologna	Previous avoidant/restrictive food intake disorder (F50.82)

13	Pavia	Major depressive disorder, single episode, in full remission (F32.5)
14	Pavia	Generalized anxiety disorder (last 6 months) (F41.1), Factitial dermatitis (L98.1)
15	Pavia	Primaryhypersomnia (F51.11)
16	Pavia	Major depressive disorder, single episode, unspecified (F32.9), Alcohol abuse, uncomplicated (F10.10), Other situational type phobia (F40.248), Body dysmorphic disorder (F45.22), Factitial dermatitis (L98.1), Primary hypersomnia (F51.11), Binge eating disorder (F50.81)
17	Pavia	Major depressive disorder, single episode, unspecified (F32.9), Premenstrual tension syndrome (N94.3), Other situational type phobia (F40.248), Separation anxiety (F93.0), Body dysmorphic disorder (F45.22), Factitial dermatitis (L98.1), Hypochondriasis (F45.21)

**nSCpatients**		
**Paziente**	**Centro di riferimento**	**SCID-5 Discreta**
18	Pisa	Criterianotmet
19	Palermo	Attention-deficit hyperactivity disorder, combined type (F90.2)
20	Palermo	Criterianotmet
21	Milano	primaryinsomnia (F51.01)
22	Bologna	Post-traumatic stress disorder (PTSD) (Subtype A: B, C, D, E +, A-) (F43.1)
23	Bologna	Panic disorder [episodic paroxysmal anxiety] (F41.0), primary insomnia (F51.01)
24	Bologna	primaryinsomnia (F51.01)
25	Bologna	Panic disorder [episodic paroxysmal anxiety] (F41.0), Post-traumatic stress disorder (PTSD) (F43.1)
26	Bologna	Major depressive disorder, single episode, in full remission (F32.5), Other eating disorders (F50.8)
27	Bologna	Otherspecifiedphobia (F40.298)
28	Pavia	Major depressive disorder, recurrent, mild (F33.0)
29	Pavia	Previous avoidant/restrictive food intake disorder (F50.82)
30	Pavia	Premenstrual tension syndrome (N94.3), Other specified phobia (F40.298), Factitial dermatitis (L98.1), Primary insomnia (F51.01), Primary hypersomnia (F51.11), Undifferentiated somatoform disorder (F45.1)

Table S8: A, B, C, D

Model Summary

Step	−2 Log likelihood	Cox & Snell R Square	Nagelkerke R Square
1	35.283[a]	0.450	0.608

a. Estimation terminated at iteration number 6 because parameter estimates changed by less than 0.001

Classification Table[a]

Observed			Predicted		
			Neuropsychiatric symptoms		Percentage Correct
			No	Yes	
Step 1	Neuropsychiatric symptoms	No	17	2	89.5
		Yes	6	22	78.6
	Overall Percentage				83.0

a. The cut value is 0.500

Variables in the Equation

		B	S.E.	Wald	df	Sig.	Exp(B)
Step 1[a]	Chorea	3.626	1.275	8.090	1	0.004	37.570
	Gender	1.650	1.122	2.162	1	0.141	5.206
	Comorbidities	−0.191	1.056	0.033	1	0.857	0.826
	Age of symptoms	0.346	0.194	3.181	1	0.074	1.414
	Treatment	−0.136	0.893	0.023	1	0.879	0.873
	Constant	−4.800	2.320	4.282	1	0.039	0.008

a. Variable(s) entered on step 1: Chorea, Gender, Comorbidities, Age of symptoms, Treatment

Variables in the Equation

		95% C.I. for EXP(B)	
		Lower	Upper
Step 1[a]	Chorea	3.088	457.134
	Gender	0.577	46.941
	Comorbidities	0.104	6.551

	Age of symptoms	0.966	2.068
	Treatment	0.152	5.030
	Constant		

a. Variable(s) entered on step 1: Chorea, Gender, Comorbidities, Age of symptoms, Treatment
